# The Relationship between Sound and Amenities of Transit-Oriented Developments

**DOI:** 10.3390/ijerph16132413

**Published:** 2019-07-07

**Authors:** Yalcin Yildirim, Diane Jones Allen, Amy Albright

**Affiliations:** 1 College of Architecture, Planning, and Public Affairs, The University of Texas at Arlington, Arlington, TX 76019, USA; 2 Transport and City Planning MSc Programme, University College London, Gower Street, London WC1E 6BT, UK

**Keywords:** TODs, train station, sound, public health, QoL, built environment

## Abstract

Experts in diverse fields have investigated sound in cities throughout the United States. This research aims to examine sound levels and determine its contributors at the transit-oriented development (TOD) station and neighborhood levels by studying selected Dallas Area Rapid Transit (DART) light rail stations. A multilevel analysis was performed to model the likelihood of TOD stations and neighborhoods affecting sound levels, controlling for station amenities, socio-demographics and built environment characteristics. Sound measurements were sampled in three time intervals with 15 min sampling over weekdays and weekends at TOD and non-TOD stations by a type II SPL meter that was mounted on a small camera tripod at a height of 1.5 m, at a distance of 1.5 m from rails and curbs. The research team found that amenities, built environmental characteristics, and neighborhood features have significant implications on sound levels at both the TOD station and the neighborhood level, which affects quality of life (QoL). TOD stations that include more amenities have a greater level of significance on sound levels. Additionally, neighborhoods with a pervasive street grid configuration, public facilities, and built environment densities are significantly associated with a likelihood of high sound levels. Conversely, higher population densities and intersection densities decrease the likelihood of a high sound level environment. These patterns provide an arena for transportation, urban, and environmental planning and policymaking to generate transformative solutions and policies.

## 1. Introduction

Noise is defined in different ways; some define it as unwanted sound, while others describe it as the combination of sounds that adversely affect hearing [1,2]. Noise, particularly environmental noise including transportation, industry, construction, and neighborhood, is often a foremost environmental issue [3]. Transportation-related noise influences more than 90% of the U.S. population, although the level of noise is not usually high enough to be considered a threat to public health [4]. There is no doubt that exposure to excessive sound levels is a part of daily urban life; however, all types of human settlements worldwide—including urban, suburban, and rural—risk exposure to potentially harmful levels of vehicle and traffic [5]. Exposure to transportation-related noise has been examined in various contexts with regard to public health concerns such as chronic diseases, hearing loss, stress, and sleeping disorders within a general context of quality of life (QoL). Before addressing the scope of the paper with regard to QoL and sound aspects, an operational definition of QoL is warranted [6]. While objective measurements of QoL consider income and crime rate variables, they do not typically reflect personal experience [7] or subjective evaluations including perceived life satisfaction based on positive or adverse feelings [8]. Regarding this category, SF-36 is a measurement of psychosocial and psychological distress and well-being [9]. Some contend, however, that subjective indicators cannot solely interpret QoL as personal welfare if it does not reflect the totality of personal circumstances [10]. Researchers have also suggested its aggregate form [11,12]. The last classification of QoL dimensions falls within discipline-specific dimensions. Public health and social science scholars have investigated the relationship between housing, neighborhood facilities, and QoL [13,14]. From health and sound perspectives, health-related quality of life (HRQOL) can be a part of this classification by measurement of the influence of a person’s health status [15]. Furthermore, the World Health Organization (WHO) Noise Guidelines are in favor of HRQOL measurements [1,16]. Considering the scope of this paper—from environmental research and urban design to urban planning—these extensive lists of QoL characteristics and definitions refer to livability, connection, mobility, personal development, community, and economic development in a broader context [17,18,19]. QoL has also been examined for neighborhood noise through various survey-based measurements [15,20,21,22], as well as in the intersection of environment, transportation and urban planning fields, such as transit-oriented developments [23]. 

Transit-oriented developments (TODs) are capturing attention globally and becoming a pivotal context in the conjunction of transportation and urban planning, particularly around light rail train stations (LRTs). LRT use in the United States has almost tripled from 1990 to 2010, with a greater increase than any other form of transit [24]. Light rail transit is a type of mass transit featured by electric powered trains performing fixed routes on the track corridors with traffic signal priority [23]. Commuters entrain at dedicated stations that are designated with various features. Those features can be related to either locating the station platform (i.e., ground level, underground, or elevated) or the facilities within the stations (i.e., restroom, seating bench etc.). 

Regarding the location of this study, the Dallas-Fort Worth (DFW) region has been a portrait of the growth and prosperity of the U.S. Sunbelt since the 1970s [25]. After being successfully officiated as a part of regional marketing and establishing a collaborative identity, Dallas and Fort Worth as one unified region, the area has experienced an increasing population growth rate [25,26]. This phenomenal challenge of addressing QoL in a rapidly growing region resulted in considering TODs on a wider “metroplex”-level scale. This study revisits the amenities of train station from TOD and non-TOD attributes through the lens of sound aspects. 

Ideal TODs provide critical livability attributes to the built environment by facilitating the use of multi-modal transit rather than driving and by increasing walking and biking [27,28,29,30,31,32]. For instance, TODs tend to generate higher-density communities with diverse land uses such as commercial, residential, and retail, and can also offer improved street connections for walking and biking circulation. Effective TODs address all age groups, creating multiple cultural, recreational, and educational facilities and opportunities [27]. These characteristics also produce distinctive sounds.

TOD-related sound mainly originates from train stations, neighborhood features such as roads, buildings, and density, and personal and transit vehicles within TODs. However, literature rarely identifies the implications of specific amenities on sound in train stations. Primary questions remain unaddressed—for instance, what other factors contribute to the emergence of noise in a TOD neighborhood? How might a TOD as a type of urban form influence these factors? Do such amenities have different effects in non-TOD and TOD neighborhoods? Little empirical evidence is presented in the literature on the mechanisms of TOD-related sound and how the built environment might affect these.

This research seeks to address this gap and investigate the relationship between TOD attributes and sound pressure levels (SPLs). Neighborhood-level data (within a radial or Euclidian quarter-mile distance, as suggested by [33,34], were used from the U.S. Census and North Central Texas Council of Governments (NCTCOG). ArcMap (10.6) tools for street and intersection-related data were utilized to examine neighborhood-level non-TOD and TOD features and SPLs at the stations [33,34].

## 2. Literature Review

### 2.1. Urban Form and Noise

Urban form refers to the physical characteristics that constitute the built environment, including the shape, size, density, and configuration of settlements [35]. Furthermore, urban form directly and indirectly affects travel behavior and air quality in addition to noise, which is the primary focus of this research [36].

Several studies have modeled noise within various urban forms. Tang and Wang (2007) assessed urban form in historic cities with various road types and different densities of intersections to investigate possible traffic noise patterns and noise levels [36]. Furthermore, Guedes, Bertolini, and Zannin (2011) conducted research that is similar in some ways to that of Tang and Wang by examining heavy and light traffic to determine whether noise levels decrease at intersections with low speed [37]. Guedes, Bertolini, and Zannin’s research findings differ, however, because they considered other factors such as pavement material, the proximity between sound and source receivers, and street configurations [37]. They also examined the physical features of urban morphology, such as compactness of place, the number of public spaces, and the physical position of buildings on the streets, and concluded that all these factors have significant impacts on noise [37]. 

Lee, Chang, and Park (2008) evaluated environmental noise through noise mapping to quantify the urban sound environment [38]. The objective of their study was to identify how the interaction between sound and urban form influences noise in an urban environment. In other research, Salomons and Pont (2012) examined the relationship between traffic noise in the built environment and urban density and form in the Netherlands [39]. Their findings indicate that building form has significant impacts on sound levels [39]. 

From another perspective, Souza and Giunta (2011) developed a model, Artificial Neural Networks, to assess sound in street environments [40]. The results of the study show that street configurations alter the sound levels in urban environments. In a broader comparative study, Wang and Kang (2011) investigated how urban morphological features affect noise in the United Kingdom and China [41]. Their study posits that urban morphology and its characteristics commonly have substantial implications for noise levels, even though the two countries studied demonstrate different urban patterns [41].

Considering more transportation-related studies, Can et al. (2008) conducted experimental research by defining noise descriptors and real urban traffic circumstances at five locations along a major road in Lyon, France [42]. They examined a one-way three-lane road with five-story buildings on both sides. The road segment investigated was crossed by six intersections and carried more than one thousand vehicles per hour. The authors aimed to understand the effects of red and green phases of traffic lights on noise propagation; however, they were not able to obtain their target findings [42]. Wu, Kang, and Zheng (2018) examined the acoustic environment of railway stations in China regarding sound field characteristics by conducting a mixed-method study in a waiting hall [43]. The authors also aimed to propose acoustical design solutions for high-speed railway stations.

### 2.2. Reviewing TOD Characteristics

As TODs are multi-disciplinary constructs, researchers from numerous disciplines, including transportation engineering, real estate, planning, and urban design, have been investigating TODs since the 1990s. TODs have experienced significant transformations as transit services continue to evolve not only in mobility options but also regarding improved technology [27]. The current concept of TODs was pioneered in the U.S. in the 1990s, but the applications and characteristics of TODs can be observed worldwide. Pojani and Stead (2015) sought to understand how urban design features could be implemented for TODs in the Netherlands [44]. In other studies, Pojani and Stead also examined TOD practices in Sweden and Austria in terms of planning policies affecting TODs [45]. This research concentrated on policy implications by performing secondary data analyses. In another study, Kong and Pojani (2017) examined the applicability of TOD principles in Beijing, China by focusing on commercial streets surrounding TOD stations [46]. Another study investigated TODs in terms of physical activity benefits relating to the walkability of TODs in Hong Kong [47]. Another study conducted an analysis in the rail stations of New York City and Hong Kong by comparing land use, socio-demographic and economic characteristics of TOD stations, concluding that the two cities have several factors in common, such as heavily used rail transportation [48].

Recent TOD facilities tend to comprise an essential set of transportation and urban design qualities and emphasize aesthetics to promote economic development. TODs typically offer multiple transportation modes including walking, biking, public transportation, and private vehicles, public facilities, such as parks, plazas, and gathering spaces, and mixed-use developments and civic engagement. A consistent body of research has explored the relationship between urban planning, transportation, and TODs [28,29,30,31,32].

The literature demonstrates that TODs influence QoL in several aspects. The fundamental purpose of TODs is to create functional places for people by integrating public transportation facilities with places where people live, work, and play [28]. Belzer and Autler concluded that well-designed streetscapes could increase mobility, the connection of neighborhood facilities, and natural amenities [49,50]. According to the literature, the benefits of TODs range from more street connectivity and multi-modal transportation to greater inclusivity for all ages and increased development [27]. Some of these outcomes, however, can generate negative externalities, such as air pollution and noise [51]. Transportation-related noise is one of the foremost types of urban noise. According to the literature, characteristics of TODs, such as mixed land uses and multi-modal transportation, are directly associated with sound levels. In other words, TODs affect a neighborhood’s sound level, which is one of the essential indicators of QoL [6].

### 2.3. TOD, Stations, and Noise

Although TODs are intended to enhance QoL through enhanced mobility, neighborhood characteristics, and multi-modal transportation, the noise levels of TODs affect QoL adversely. The literature confirms that noise has psychological impacts (the annoyingness or pleasantness of sound), mental effects (sleeping disorders, anxiety) or both [52]. The mechanisms of noise exposure may cause critical problems such as noise-induced hearing loss, cardiovascular disease, and sleep [1]. TODs are considered to be essential factors in ideal urban environments; however, traffic-related noise poses an explicit threat to QoL [53]. 

A number of studies have investigated the relationship between TODs and noise. One study examined components of TODs such as the indoor and outdoor acoustic environment of metro stations [54]. Another study shows that people are reluctant to move into TODs because they are concerned about noise and vibrations [55]. For example, houses within the first lots adjacent to TOD stations are typically not sold as quickly as other lots [55] located within TODs. In another study, Renne (2009) considered noise as a QoL indicator and performed interviews to record TOD residents’ perception of noise [56]. More than 40% of the participants considered TODs noisy locations, while 38% believed they were quiet [56]. Thus, people living near TODs have mixed opinions about the effects of sound on their daily lives.

Other studies examined the negative externalities of sound in TODs, which generate noise related to driving, even though people near TODs generally drive less [57]. Studies have calculated the costs associated with these negative externalities. Based on one such computation, the total daily cost of TOD benefits in the city of Jersey City is approximately $20,000, with only $14 in negative externalities for noise [57]. Since decreased motor vehicle numbers correlate with lower sound levels, TOD sites with facilities such as bike paths or pedestrian ways enhance the streetscape and lead to reduced traffic speed and traffic-related sounds [58]. Applying expanded traffic noise investigation in San Francisco with different types of vehicles in urban communities, one study demonstrates that enhancing walking, biking, car sharing, public transit, and home office working contributes to reduced urban noise and improves QoL [59]. 

Regarding the relationship between noise, train stations and tunnels in terms of public health, Xie, Peng, Wang, and Zhang conducted experiments in tunnels to examine the effects of high-speed rails on hearing [60]. The authors found that acoustic discomfort occurred when a train passed in the middle of the tunnel [60]. In a similar study, Maclachlan, Ögren, van Kempen, Hussain-Alkhateeb, and Persson Waye (2018) examined the relationship between annoyance and rail vibrations while considering neighborhood distance to analyze public health implications by using a self-reported questionnaire of 6894 persons in Sweden [61]. The findings of the study highlight that there is an association between the distance from a rail transit station and annoyance from noise [61]. In another study, Mao et al. (2019) examined more broadly the relationship between underground transportation and environmental quality, including thermal environment, air quality, lighting environment, and acoustic comfort [62]. Their findings regarding acoustic environment show that subway platforms are noisier when a train leaves compared to when it arrives at platforms [62].

Despite the correlation between TODs, stations, and sounds, little empirical evidence exists at the station and neighborhood scales regarding how sound might be affected by station facilities and TOD characteristics. This is primarily due to the lack of data available and the difficulty of sound pressure level data collection at both scales. Therefore, by addressing the shortcomings of existing studies and the need for more sound-related studies, this study examines the nexus between SPLs and TOD and non-TOD station amenities. 

## 3. Research Methods

### 3.1. Research Process and Variables

To assess the relationship between the amenities of TOD stations and sound, it is necessary to first control other factors of amenities and neighborhoods. To do this, the research team aimed to define study locations as the first phase of the research method. As an initial process, cluster analysis was used to classify rail station areas based on density, diversity, land use, and walkability, built environment features extensively suggested by the literature [63,64]. The research team included various neighborhood characteristics based on QoL and health variables, which included jobs within a quarter-mile radius and neighborhood amenities including entertainment, education, recreation areas, libraries, shopping centers, healthcare, population density, employment, and modes of transportation for each station. In the second phase of the study selection, a qualitative process determined the final study locations.

### 3.2. TOD Station Area Definition

TODs are expected to include pedestrian-friendly urban design and employment density. In light of this, the literature suggests that the proximity of TODs to the station range from a quarter mile to one and a half miles. To obtain more accurate sound samples, this study used the suggested TOD area of a quarter mile [28]. Drawing on similar methodologies performed in previous studies [29,63], based on the two-phase suitability analysis, the researchers identified the TOD and non-TOD stations to perform further advanced comparative analyses (Table 1). Since the literature primarily highlights three built environment factors that indicate whether a station performs as a TOD, the research aimed to group existing stations based on built environment performance. Thus, data for the three characteristics were collected and analyzed at each station beforehand, and a normalization procedure was performed to standardize each built environment factor between 0 and 1. The sum of population and employment refers to the total population added to the number of jobs based on the U.S. American Community Survey, while land use density distinguishes various land use categories such as retail, education, and residential, and ranges from 0, indicating and entirely single-use area, to 1, where the land is evenly divided among various land uses. Finally, intersection density is the sum of all types of intersections within each station area [29,63].

**Figure 1:**
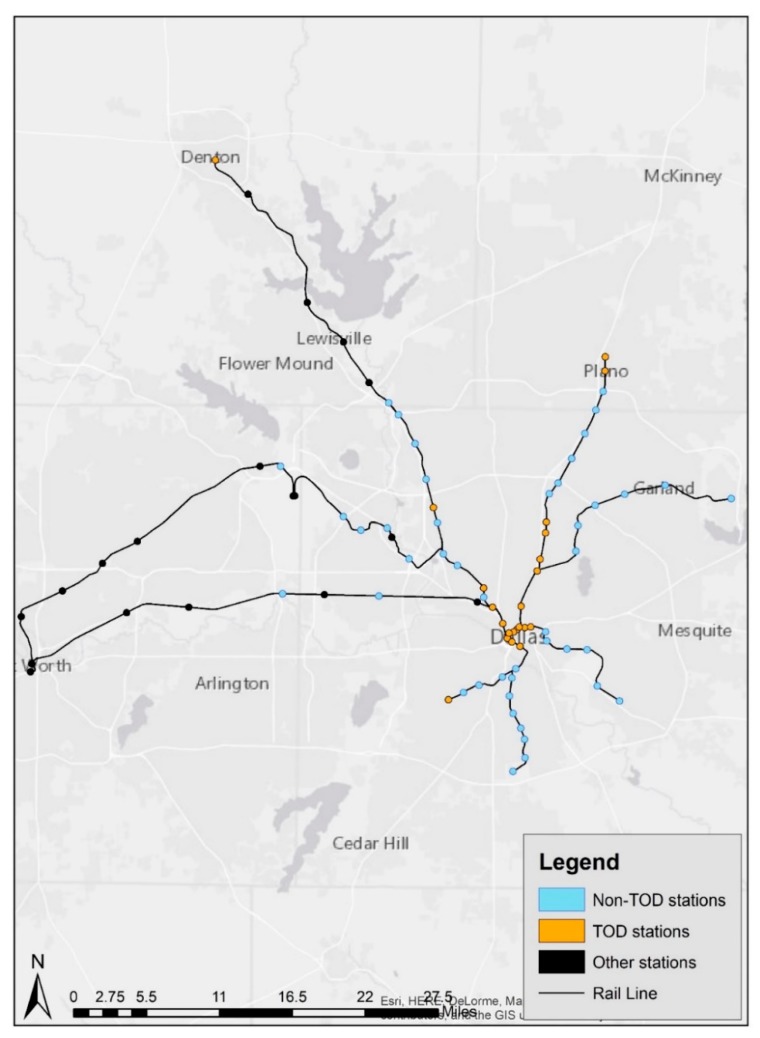
Study locations (Source: North Central Texas Council of Governments (NCTCOG), 2018).

Based on the study selection process, Figure 1 illustrates TOD stations and study locations based on the selection criteria (yellow dots refer to TOD study locations and blue dots represent non-TOD stations). Most of the regions’ TOD stations were constructed in 1996 and 1997, and TOD features were implemented through various economic and development incentives, such as tax increment financing (TIF), to date. In addition, TOD construction emerged from different motivations. For instance, proximity to the central business district made stations such as West End, Akard, St. Paul, and Pearl inherently logical for development as TODs, while developers’ attention and initiations occurred at Mockingbird, Downtown Plano, Park Lane, and Cedars stations. Additionally, various factors such as medical district and hospital effects (Baylor Medical station), and business hub locations (City Place, Victory and Market Center stations), which are associated with population, employment, land use diversity, and intersection quantities, were instrumental to TOD development for these stations [65,66]. Considering the non-TOD stations, the normalized cluster analyses show that the sum of population, employment, land use diversity, and intersection density remains low. Non-TOD stations also differ from other stations in the figure as they are operated by Dallas Area Rapid Transit (DART), whereas other stations serve heritage and commuter rail services (TEX Rail and Trinity Railway Express) [67]. As an explanatory note, although the region includes 84 train stations, 17 stations with different rail infrastructure, including heritage railroads and commuter rail, were excluded in order to standardize study locations. This resulted in the selection of 67 light rail train stations (Figure 1). After identifying these stations, the research team identified 22 stations as TODs and 45 stations as non-TODs in order to obtain a standardized comparison framework to observe the implications of sounds on TODs and non-TODs. 

The researchers performed sound pressure level (SPL) measurements with the A-weighted (dBA), considered a Level 1 dependent variable, at each study station at different time intervals and days of the week (Table 2). SPL measurements were performed on selected days from October 2018 to March 2019 at 10:00, 13:00, and 16:00. Furthermore, measurements were recorded for both weekdays and weekends to control for differences in other variables, including ridership effects across these time frames. 

**Figure 2:**
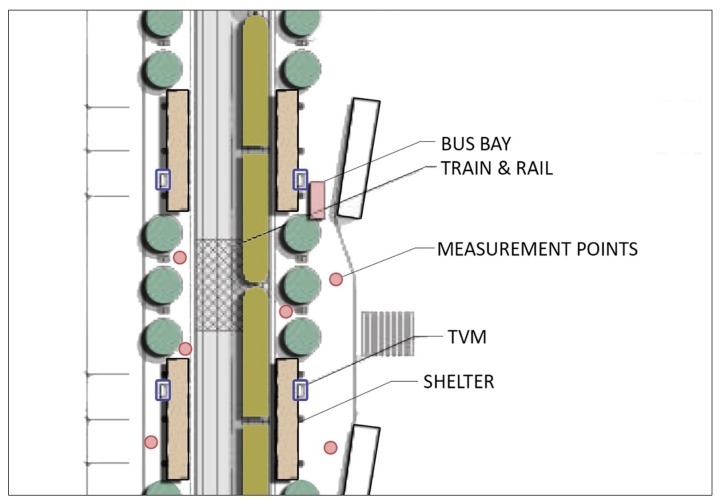
Sample sound measurement approach (Source: Dallas Area Rapid Transit (DART), 2008).

For the sound pressure level measurement, Landtek Instruments Professional Digital Sound Level meter 30–130 dB with the capacity to weight frequencies to either the A, C, or F (flat) scale with windshield (to reduce the effects of wind and air movements in the microphone) and Bluetooth equipment was used. Sound levels were measured at a standard 5 ft (1.5 m) distance from the ground, rails, and curbs and 10 ft (3 m) distance to station entry plazas if available (i.e., Mockingbird Station) and front, middle, and rear sections of the platforms [37]. As Figure 2 illustrates, sound level measurements took place at six randomly selected locations on each train station platform to obtain maximum sound samples based on the standardized approach. Since train stations are located at side platforms or center platforms for all study locations, the sampling approach was arranged according to these factors. For stations with side platforms, the sampling was performed on both sides (three on each side), while stations with center platforms followed the six sampling points at the stations. Eventually, the researchers obtained a total of 402 sound samples in 67 study locations. Measurements were aimed to prevent any echo from the entrance region, and all values were recorded in decibels (dBA). Average quiet residential areas tend to register at approximately 40 dB, freeway traffic at 70 dB, and a car horn at 110 dB [68].

The model of sound pressure level meter was IEC651 Type 2, sound level meter standards by International Electrotechnical Commission, ANSI S 1.4. The L_eq_ fifteen-minute method was performed for this research to identify variations in sound over time because of its significance as a reference sign of sound investigation in addition to its implications on people [69,70,71]. The measurements were gathered by the researchers in periods that did not experience extreme weather conditions, such as strong wind or heavy rain. Further, the measurements were conducted when there were no trains in or heavy construction machines around the stations to avoid excessive SPL and homogenize the measurements across the TOD stations as the research team encountered several outlying circumstances: seven instances of rainy or windy weather conditions, two emergency situations (ambulance and police sirens), and two instances of excessive construction noise around the stations (Deep Ellum and West End).

In addition to sound measurements, various attribute data were collected for both station and neighborhood levels to investigate the implications of such features on sound pressure levels. To control for built environment at both levels, the research first identified station amenity-related variables (Table 2). Since train stations exist as public or semi-public environments, the research aimed to include as many station-related amenities as possible and examine the relationship between sound and each amenity individually. As a note on variables, the research team removed the presence of public art at the stations, as all stations except for one displayed public art. Variables that were considered, as suggested by literature [72,73,74,75,76,77,78,79,80,81], included structural features like walls within the stations, restrooms, information centers, ticket offices, crew rooms, and map boxes to observe whether they emitted sound throughout the stations or not [72,73,74,75]. Also, crew rooms, restrooms, shelters, benches, windscreens, bus bays, and trash receptacles are regularly cleaned by custodial staff in the study locations [76]. Therefore, light or heavy cleaning may result in changes to sound levels. Trash receptacles can also produce additional sound from the disposal of rubbish. Furthermore, an average typical front-end garbage truck produces sound levels between 65 dBA and 94 dBA [77]. Ticket vending machines (TVMs) or ticket offices are also examined in several studies regarding sound and station facility relationships [38,78]. The number of parking spaces is considered to assess the relationship between sound levels and personal vehicle and ride-sharing services (Uber or Lyft) adjacent to the stations [79,80]. Bike facilities are important features of TOD stations as a part of multi-modal transportation [72,80]. Stations designed with bike lockers encourage frequent and high usage of bicycles as a mode of transportation [80]. In other words, biking and more bike-related amenities tend to correlate to less personal automobile usage around the stations. Transit ridership is one of the main goals of transit agencies on commuting services for individuals [43,72,81]. Facility type is another significant variable in the literature, particularly for sound implications. Several studies attempted to examine stations located at ground level, elevated, or underground to examine the acoustic features of train stations in terms of reverberation, finishing materials, tunnels or elevated materials aspects [72,75]. The analyses also controlled for numerous built environments, socio-demographic, and geospatial variables at the neighborhood scale (Table 2). The most frequently highlighted variables in the literature on urban noise at neighborhood level are street connectivity, traffic speed, population, employment, neighborhood amenities and presence of grid street layouts [82,83,84,85,86]. These data were collected by various local, regional, and national data sources. As Table 3 shows, Level 1 independent variables were obtained from Dallas Area Rapid Transit (DART), the North Central Texas Council of Governments (NCTCOG), and site visits. Level 2 variables were extracted from the NCTCOG and the U.S. Census. Speed limit represents the average speed limit of the road segments in each study location and street density is the sum of the streets within each study area at a quarter-mile radius. The number of jobs was extracted from the NCTCOG and the U.S. Census American Community Survey 5-year estimates during the period 2010–2014. Since these data are available at a half-mile radius, they were first summed to find the activity density and divided into two in order to examine the activity density at a quarter-mile buffer.

### 3.3. Statistical Analysis

**Figure 3:**
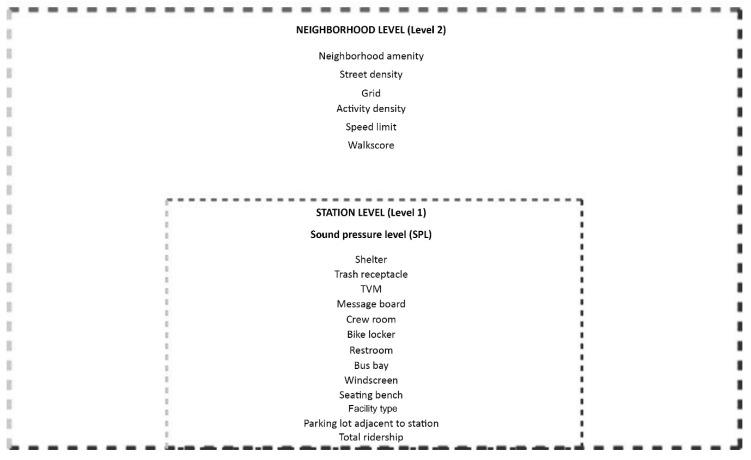
Conceptual hierarchical linear modeling (HLM) Nesting Structure for the Variables.

Sound pressure level (SPL) is the dependent variable in this study. As this is a continuous variable, regression modeling can be used. As shown in Figure 3, the data used in this analysis demonstrate a “nested” structure and need to be analyzed accordingly. Since all neighborhoods studied surround transit stations, they share characteristics of the stations, such as street connectivity. Therefore, such characteristics could not be considered independent. The nesting structure is inclined to generate dependence among cases, violating the independence conjecture of ordinary least squares (OLS) regression. Standard errors of regression coefficients connected to neighborhood characteristics relying on OLS will subsequently be miscalculated, and therefore regression coefficients will not be efficient [87].

Hierarchical modeling surpasses the limitations of OLS, computing the dependence among cases and generating more precise coefficient and standard error estimates. In the context of a hierarchical model, each level in the data profile is represented by its configuration, and these configurations are statistically related. Hierarchical linear modeling (HLM) computes for dependence among samples; in our model, this is the dependence of neighborhood levels on the characteristics of the TODs. Hierarchical linear modeling demonstrates a parallel pattern to regression modeling while it operates, as with a multi-level data configuration. Thus, hierarchical linear models were estimated for the various outcomes of sound pressure levels. 

In this research, the sound pressure levels (SPLs) of the stations were processed on neighborhood characteristics in the Level 1 configuration. The intercepts and coefficients of Level 1 models were operated on neighborhood characteristics in Level 2 models. Essentially, since different models were projected, only the intercepts randomly varied, whereas all the regression coefficients were performed as fixed. These are denoted as “random intercept” models. Later, regression coefficients were agreed to vary across higher level units randomly, and interactions within levels were computed. These are entitled random coefficient models. In order to interpret the relationship between sound levels and the models, sets of statistical analyses were performed. The statistical analysis software IBM SPSS Statistics for Windows, version 25 (IBM Corp., Armonk, NY, USA) and Scientific Software International HLM for Windows, version 7.03 (Scientific Software International, Inc. Skokie, IL, USA) was utilized to analyze the correlation tests and multi-level linear modeling, respectively. Before performing these tests, the research performed all variance inflationary factor (VIF) values of the multicollinearity test that is within 1 and 10, with a maximum value of 7.73 and mean value of 2.87, indicating acceptable levels of collinearity [88].

## 4. Findings and Results

Pearson correlation coefficient was used for the measurement of linear dependence between sound and other variables (Table 4). The correlations between sound levels and the indicators are shown in Table 3. Overall, Level 1 variables of non-TOD stations are significantly associated with sound levels that are significantly correlated with the seating benches, message boards, ticket vending machines (TVMs), and shelters (*p* < 0.05). When we look at the same scale indicators of TODs, message boards and facility types are significantly associated with the sound levels. Level 2 indicators also include a relatively significant correlation with amenities for non-TOD stations.

The coefficients of all variables show the expected signs and many of them are significant at the 0.05 level (Table 5 and Table 6). The significant variables are in bold font. The variables that control for the non-TOD station amenities are significant at the various probability levels; however, they do not show significance for the model. The odds of sound levels at non-TOD stations represent a positive and robust relationship with station amenities. This illustrates a causal correlation between the station amenities and sound levels.

The number of TVMs and bus bays in non-TOD stations significantly increases the likelihood of the location having louder sound levels, while the number of seating benches decreases the odds of a non-TOD station having louder sound levels. The sound level of the non-TODs at a quarter-mile distance is also highly significant at the neighborhood level. Controlling the covariates, a neighborhood with a higher speed limit is more likely to have higher sound levels in the neighborhoods (Table S1). 

Considering the TOD stations, there is a remarkable variance from non-TOD stations. Almost all Level 1 variables, except crew room and bus bays, show varying degrees of significant relationships with sound levels. The number of shelters in a TOD station significantly decreases the likelihood of the location having louder sound levels. Bike lockers, restrooms, and seating benches also significantly reduce the likelihood of louder sounds. Furthermore, message boards, trash receptacles, and TVMs significantly increase the likelihood of the location having louder sounds. This suggests that amenities which emit sound themselves, such as TVMs and message boards, increase sound levels in TOD stations, whereas amenities that represent passive interaction, such as seating, or structure-related amenities, including restrooms, bike lockers, and shelters, are more likely to have neutral or negative tendencies for sound levels. Moreover, stations located on aerial platforms include higher sound levels compared to stations with at-grade rail platforms.

Regarding neighborhood-level variables, the number of neighborhood amenities significantly increases the likelihood of observing louder sound samples. Similarly, street density, and particularly grid street schemes, of TOD stations also increase sound levels. Conversely, a higher walkscore decreases the probability of a TOD neighborhood having a louder sound level. This is due to higher walkscores representing more walkable neighborhoods and potentially lower vehicle noise. Surprisingly, activity density, including population and employment, around the TOD stations shows a significant relationship with sound; however, its coefficient is almost zero. Thus, the TOD neighborhood areas influence sound levels, corresponding to the built environment components of the neighborhood but not significantly to the socio-demographic characteristics (Table S1).

## 5. Discussion

The aim of this research was to examine the amenities of train stations and surrounding neighborhood areas associated with sound and to assess such characteristics in regard to transportation and urban planning policies. To achieve this, the research team investigated station amenities and the built environmental and socio-demographic characteristics of neighborhoods surrounding non-TOD and TOD stations to explain likely patterns of sound levels. Other scholars have concentrated on the effects of socioeconomic characteristics, particularly age, gender, and education level [89,90,91,92] on sound levels. However, the scope of this research accounts for variables at two levels of geography, namely, at the station and neighborhood levels, and controls for neighborhood differences using hierarchical modeling.

While socio-demographic characteristics, such as population density and employment density, do not attribute implications on sounds, amenities of stations and built-environment-related factors have effects on the likelihood of a quiet or noisy TOD or non-TOD station environment. Neighborhoods with more built environment characteristics, including neighborhood amenities such as parks and libraries, as well as dense street and road connections, are more likely to generate higher sound level acoustic environments. A dispersed built environment form, higher street density, higher speed limits, and more grid street configurations are the primary drivers of higher sound levels, particularly in TODs, which confirms other scholars’ findings [86,93].

Additionally, both station and neighborhood amenities of non-TOD stations have fewer implications on the sound level compared to TOD stations in this research. This evidence suggests the complexity of various components of TODs that have implications on sound propagation compared to non-TODs. As these findings demonstrate distinct features of TODs and their amenities, the researchers urge the adoption of policies that consider the effects of noise on buildings as cities consider building more TODs. Even though monitoring sound levels at each station may be difficult, a general consideration of noise level allowances at the neighborhood or station level may help to improve residents’ QoL. Hence, applying a noise ordinance for TODs and non-TODs using guidelines such as Caltrans’ “Traffic Noise Analysis Protocol for New Highway Construction, Reconstruction and Retrofit Barrier Projects” may reduce certain noise levels to improve QoL [94].

As sound is highly associated with public health, quality of life (QoL), perceptions, the built environment, and amenities, it is a crucial factor for consideration by engineers, planners, transit authorities, and local city officials. Increasing the amenities, land use varieties, street densities, and speed limits of neighborhoods is likely to increase the sound levels. Before enacting policies related to these aspects, decision-makers might investigate the perception of increasing or decreasing sound levels for residents. 

The differences observed between TOD and non-TOD stations may, however, be caused by the context or limitations of the research. Moreover, since sound is affected by many station and built-environment characteristics, the research inherently includes generalizability issues, as other station amenities and neighborhood features in different cities or countries could affect sound implications. Also, since several variables were calculated through the ArcMap tools and secondary data sources, some of the assumptions and sampling are not avoidable for this research, as well as the SPL meter calibration procedure. Nonetheless, the study aimed to investigate the relationship between TOD and non-TOD station characteristics with particular focus on built environment characteristics. Further studies may examine this by including more socio-economic variables, urban design features, as well as studying areas surrounding stations to evaluate the effects of distance on sound levels by conducting a survey-based quality of life measurement of sound implications to clarify the effects of high sound levels at TODs. 

## 6. Conclusions

Cities are increasingly considering implementing TODs to improve the QoL of residents. While TODs promote healthy living environments around transit centers that serve many people with rail stations and facilities, they threaten public health in terms of the acoustic implications of station amenities. This research found that amenities of TOD and non-TOD stations, among various indicators from the station and neighborhood levels, have implications on acoustic realms. In addition to station amenities, neighborhood characteristics also affect sounds.

The research team identified that both station- and neighborhood-level indicators play a significant role in contributing to lower or higher sound levels, and this pattern most notably occurs in TOD stations. This may be caused by the characteristics of TODs, which encourage dense population, activity, land uses, and more connective street layouts for multi-modal transportation. Another critical implication for the planning and transportation fields is that each TOD station includes unique sound sources and characteristics. This “locality” compels more consideration for the planning and design of each TOD. Therefore, specific design and planning efforts should keep sound in mind when addressing the context of a TOD area. This research suggests that local authorities considering the implementation of TODs perform surveys to acquire a better understanding of local preferences. However, it is imperative to keep in mind that, as Shephard, Welch, Dirks, and Mathews (2010) discussed, sound pressure level is not always consistent with noise annoyance, and SPLs do not provide information relevant for acoustic comfort or noise annoyance [15]. So, considering QoL perspectives, various further research directions could be followed to draw a more robust conclusion [95]. However, the goal of this study is similar to the DYNAMAP Project, aiming to generate an acoustic impact map to assess and manage noise and to provide an urban case study from a different point of view [96].

Understanding the characteristics of sound and sound environments is critical for urban planners, landscape architects, transportation planners, and policymakers to develop policies that manage sound level environments in TODs. One of the goals of current policies, such as noise abatement, has been to incorporate sound level management into stations and TODs. This research could be applied by approaching TOD station management and surrounding neighborhoods to present the study’s findings in order to promote participatory planning, so that the complexity of the TOD concept may help to manage Not in My Backyard (NIMBY) concerns of neighborhood residents. 

To further support acoustic experts, urban planners, urban designers, public health experts, and policymakers focusing on this issue, many of the largest U.S. metropolitan areas, including Denver, Portland, Minneapolis, and Dallas, have been adapting TOD concepts to create transit-friendly urban hubs, in recent decades [97]. By managing noise concerns at the station and neighborhood level, health outputs may improve and TODs may better serve their residents and visitors. Moreover, strategies that integrate urban design, public health, technology, and regulation within a collaborative arena of planning, policy making, and acoustics hold promise for increasing health outputs of TODs, including the potential to address noise concerns.

## Figures and Tables

**Table 1:** Variables for station criteria.

Station Type	Activity Density	Land Use Density	Intersection Density	Total
	Sum of Population and Employment	Land Use Coverage Categories	Sum of All Type of Intersection	Total Normalization Score
Non-TOD	0.19	0.07	0.15	0.40
TOD	0.44	0.29	0.56	1.29

**Table 2:** Variable review summary on both train station and neighborhood levels.

Author/s	Train Station-Related Variable	Method	Location
Dinno et al.	Partial or full enclosure of a station and rail	SPL measurement	San Francisco, Bay Area Rapid Transit (BART)
Yao et al.	Platform design, station size, train platform v. train inside v. combined effects	SPL measurement	Toronto, Canada
Yao et al./U.S. DOT Federal Railroad Administration	Bikes, bicycle racks, bicycle parking lots, bikers	SPL measurement	Toronto, Canada
Yao et al./Loukaitou-Sideris and Schaffer/Houston et al./Shimokura and Soeta	Structural platform includes wall, lateral wall, or similar material in the stations	SPL measurement	Toronto, Los Angeles, Japan
Shimokura and Soeta	Architectural elements of the stations—shelter and roof	SPL measurement	Japan
Shimokura and Soeta	Reflection from the structural elements in the station such as message board	SPL measurement	Japan
Yao et al./Shimokura and Soeta	Platform facility type (whether the station is elevated, above or underground)	SPL measurement	Toronto, Japan
Wu, Kang, and Zheng/Su and Caliskan	Ticket office, ticket machine, kiosk, customer information	SPL measurement and Survey for Acoustic Comfort	China, Turkey
Wu, Kang, and Zheng	Waiting hall including seating bench	SPL measurement and Survey for Acoustic Comfort	China
California High Speed Rail Authority/U.S. DOT Federal Railroad Administration	Parking structure, kiss-n-ride passenger drop-off adjacent to a train station	SPL measurement	California
Yao et al.	Ridership	SPL measurement	Toronto, Canada
Dinno et al./Wu, Kang, and Zheng/Loo, Chen, and Chan	Population, sociodemographic features, and ridership	SPL measurement	San Francisco, Bay Area Rapid Transit (BART)
Dinnoo et al./Gherson et al./Shimokura and Soeta/Loukaitou-Sideris and Schaffer	Speed limit	SPL measurement	San Francisco, New York City, Japan, Los Angeles
Jacobs/Gozallo and Morillas	Grid	SPL measurement	Various cities around the world, Chile
Wu, Kang, and Jian/Han et al.	Street and intersection density	Prediction modelling, Digital projecting	China, China

**Table 3:** Variables used to explain the odds of sound pressure level (SPL) in the transit-oriented developments (TODs).

Variables		Data Sources
**Level 1 Dependent variable**		
SPL	Sound pressure level/s (at each station)	The authors
**Level 1 Independent variables (Station Level)**		
Seat	Number of seats	DART, site visit
Board	Number of message boards	DART, site visit
Trash	Number of trash receptacles	DART, site visit
Shelt	Number of shelters	DART, site visit
Crew	Number of crew rooms	DART, site visit
Busb	Number of bus bays	DART, site visit
Winds	Number of windscreens	DART, site visit
BL	Number of bike lockers	DART, site visit
TVM	Number of TVMs	DART, site visit
Ride	Number of riders	DART, NCTCOG
PLot	Number of parking lots of stations	DART, Google Earth
Facility type	Whether the rail is on the grade rail or aerial platforms (dummy)	DART, Google Earth
**Level 2 Independent variables (Neighborhood Level, a quarter-mile)**		
Sden	Street density (linear)	ArcGIS, NCTCOG
Gden	Grid density (binary)	ArcGIS, NCTCOG
SpLim	Speed limit (linear)	ArcGIS, NCTCOG
Amen	Number of amenities (linear)	ArcGIS, Google Earth
ActDen	Sum of jobs and population (linear)	ACS 20162010-2014, NCTCOG
Wscore	Walkscore (linear)	Walkscore.com

**Table 4:** Pearson correlation coefficients between sound measurements and indicators.

Non-TOD Level 1	S	TR	TV	MB	CR	BL	RR	BB	WS	Sb	F	PL	R
SPL	0.296 *	0.002	0.316 *	0.343 *	−0.187	−0.078	−0.126	0.145	0.178	−0.383 **	0.221	−0.006	0.066
Non-TOD Level 2	AM	Sden	G	Aden	Sp	WS							
SPL	0.296 *	0.127	−0.206	-0.210	0.248	−0.056							
TOD Level 1	S	TR	TV	MB	CR	BL	RR	BB	WS	Sb	F	P L	R
SPL	−0.269	0.160	0.289	0.475 *	−0.022	0.059	−0.055	0.247	−0.278	−0.047	0.524 *	0.157	0.106
TOD Level 2	AM	Sden	G	Aden	Sp	WS							
SPL	0.366	−0.060	0.078	−0.126	0.356	−0.040							

* Correlation is significant at the 0.05 level (two-tailed); ** Correlation is significant at the 0.01 level (two-tailed). S: Shelter, TR: Trash receptacles, TV: Ticket vending machine, MB: Message board, CR: Crew room, BL: Bike Lockers, RR: Restrooms, BB: Bus bays, WS: Windscreen, Sb: Seating bench, F: Facility, PL: Parking lot, R: Ridership, AM: Amenities, Sden: Street density, G: Grid, Aden: Activity density, Sp: Speed limit, WS: Walkscore.

**Table 5:** Hierarchical linear modeling of log odds of sound levels in non-TOD stations.

	Coefficient	Standard Error	t-ratio	*p*-value
Constant	42.816	6.518	6.568	<0.001
Level 1				
Shelters	−0.439	0.303	−1.450	0.157
Trash receptacle	−0.004	0.142	−0.035	0.973
TVM	2.389	0.897	2.662	0.012
Message board	2.108	1.384	1.523	0.138
Crew room	−2.686	1.596	−1.682	0.102
Bike lockers	−0.495	0.427	−1.161	0.254
Restrooms	−1.017	0.845	−1.203	0.238
Busbays	0.807	0.346	2.329	0.026
Windscreens	−0.024	0.165	−0.146	0.885
Seating bench	−0.098	0.044	−2.192	0.036
Facility	2.305	2.027	1.137	0.264
PLot	−0.001	0.002	−0.545	0.590
Rider	−0.000	0.000	−0.667	0.509
Level 2				
Amenity	0.263	0.285	0.924	0.361
Street den.	0.012	0.202	0.059	0.953
Grid	−0.823	1.226	−0.671	0.506
Activity den.	0.000	0.000	0.422	0.676
Speed	0.495	0.092	5.377	<0.001
Wscore	−0.017	0.027	−0.642	0.525

**Table 6:** Hierarchical linear modeling of log odds of sound levels in TOD stations.

	Coefficient	Standard Error	t-ratio	*p*-value
Constant	72.547	6.635	10.934	<0.001
Level 1				
Shelters	−2.168	0.246	−8.809	<0.001
Trash receptacle	0.508	0.124	4.071	0.003
TVM	2.061	0.530	3.889	0.004
Message board	3.406	0.213	15.953	<0.001
Crew room	−1.556	1.503	−1.036	0.327
Bike lockers	−1.710	0.297	−5.753	<0.001
Restrooms	−1.787	0.348	−5.125	<0.001
Busbays	−0.559	0.294	−1.903	0.089
Windscreens	1.295	0.100	12.914	<0.001
Seating bench	−0.076	0.025	−3.048	0.014
Facility	14.202	1.025	13.843	<0.001
PLot	0.021	0.002	10.622	<0.001
Rider	0.000	0.000	−4.821	<0.001
Level 2				
Amenity	0.877	0.129	6.784	<0.001
Street den.	0.206	0.024	8.412	<0.001
Grid	13.457	1.007	13.353	<0.001
Activity den.	0.000	0.000	3.226	0.006
Speed	0.511	0.059	8.596	<0.001
Wscore	−0.624	0.120	−5.198	<0.001

## Supplementary Materials

The following are available online at https://www.mdpi.com/1660-4601/16/13/2413/s1, Table S1: Site measurements and variables.

## References

[1] Berglund B., Lindvall T., Schwela D.H. (1999). Guidelines for Community Noise.

[2] Stephen A.S., Mark P.M. (2003). Noise pollution: Non-auditory effects on health. Br. Med Bull..

[3] Kang J. (2017). From dBA to soundscape indices: Managing our sound environment. Front. Eng. Manag..

[4] U.S. Department of Transportation, Bureau of Transportation Statistics, National Transportation Noise Map; U.S. Census Bureau, 2014 American Community Survey 5-year Estimates; National Institute on Deafness and Other Communication Disorders, I Love What I Hear! Common Sounds. https://www.nidcd.nih.gov/health/i-love-what-i-hear-common-sounds.

[5] Firdaus G., Ahmad A. (2010). Noise Pollution and Human Health: A Case Study of Municipal Corporation of Delhi. Indoor Built Environ..

[6] Lee R.J., Sener I.N. (2016). Transportation planning and quality of life: Where do they intersect?. Transp. Policy.

[7] Sirgy M.J., Michalos A.C., Ferriss A.L., Easterlin R.A., Patrick D., Pavot W. (2006). The quality-of-life (QOL) research movement: Past, present, and future. Soc. Indic. Res..

[8] Diener E. (2000). Subjective well-being: The science of happiness and a proposal for a national index. Am. Psychol..

[9] Lins L., Carvalho F.M. (2016). SF-36 total score as a single measure of health-related quality of life: Scoping review. SAGE Open Med..

[10] Felce D., Perry J. (1995). Quality of life: Its definition and measurement. Res. Dev. Disabil..

[11] Bowling A., Gabriel Z., Dykes J., Dowding L.M., Evans O., Fleissig A., Banister D., Sutton S. (2003). Let’s ask them: A national survey of definitions of quality of life and its enhancement among people aged 65 and over. Int. J. Aging Hum. Dev..

[12] Sarch A.F. (2012). Multi-component theories of well-being and their structure. Pac. Philos. Q..

[13] Sirgy M.J., Cornwell T. (2002). How neighborhood features affect quality of life. Soc. Indic. Res..

[14] Bize R., Johnson J.A., Plotnikoff R.C. (2007). Physical activity level and health-related quality of life in the general adult population: A systematic review. Prev. Med..

[15] Shepherd D., Welch D., Dirks K.N., Mathews R. (2010). Exploring the relationship between noise sensitivity, annoyance and health-related quality of life in a sample of adults exposed to environmental noise. Int. J. Environ. Res. Public Health.

[16] (2009). Night Noise Guidelines for Europe.

[17] van Kamp I., Leidelmeijer K., Marsman G., de Hollander A. (2003). Urban 947 environmental quality and human well-being: Towards a conceptual framework and 948 demarcation of concepts; a literature review. Landsc. Urban Plan..

[18] Jacobs J. (1961). The Death and Life of Great American Cities.

[19] Smith T., Nelischer M., Perkins N. (1997). Quality of an urban community: A framework for understanding the relationship between quality and physical form. Landsc. Urban Plan..

[20] Neitzel R.L., Gershon R.R.M., McAlexander T.P., Magda L.A., Pearson J.M. (2012). Exposures to Transit and Other Sources of Noise among New York City Residents. Environ. Sci. Technol..

[21] Nitschke M., Tucker G., Simon D.L., Hansen A.L., Pisaniello D.L. (2014). The link between noise perception and quality of life in South Australia. Noise Health.

[22] Botteldooren D., Dekoninck L., Gillis D. (2011). The influence of traffic noise on appreciation of the living quality of a neighborhood. Int. J. Environ. Res. Public Health.

[23] Boorse J.W. (2001). This is Light Rail Transit.

[24] Neff J., Dickens M. (2012). Public Transportation Fact Book Appendix A: Historical Tables.

[25] Hanlon B., Short J.R., Vicino T.J. (2010). Cities and Suburbs: New Metropolitan Realities in the US.

[26] North Texas Commission History. https://ntc-dfw.org/about-us/history/.

[27] Curtis C., Renne J.L., Bertolini L. (2009). Transit Oriented Development: Making It Happen.

[28] Calthorpe P. (1993). The Next American Metropolis: Ecology, Community, and the American Dream.

[29] Ewing R. (1999). Pedestrian- and Transit-Friendly Design.

[30] Dittmar H., Poticha S., Dittmar H., Ohland G. (2004). Defining Transit-Oriented Development: The New Regional Building Block. The New Transit Town: Best Practices in Transit-Oriented Development.

[31] Jacobson J., Forsyth A. (2008). Seven American TODs: Good practices for urban design in Transit-Oriented Development projects. J. Transp. Land Use.

[32] Ewing R., Bartholomew K. (2013). Pedestrian & Transit-Oriented Design.

[33] Guerra E., Cervero R., Tischler D. (2012). The Half-Mile Circle: Does It Best Represent Transit Station Catchments?. Transp. Res. Rec..

[34] Atkinson-Palombo C., Michael J.K. (2011). The Geography of Advance Transit Oriented Development in Metropolitan Phoenix, Arizona, 2000–2007. J. Transp. Geogr..

[35] Dempsey N., Brown C., Raman S., Porta S., Jenks M., Jones C., Bramley G., Jenks M., Jones C. (2010). Elements of Urban Form. Dimensions of the Sustainable Cities.

[36] Tang U.W., Wang Z.S. (2007). Influences of urban forms on traffic-induced noise and air pollution: Results from a modelling system. Environ. Model. Softw..

[37] Guedes I.C., Bertoli S.R., Zannin P. (2011). Influence of urban shapes on environmental noise: A case study in Aracaju Brazil. Sci. Total Environ..

[38] Lee S., Chang S., Park Y. (2008). Utilizing noise mapping for environmental impact assessment in a downtown redevelopment area of Seoul, Korea. Appl. Acoust..

[39] Salomons E.M., Pont M.B. (2012). Urban traffic noise and the relation to urban density form and traffic elasticity. Landsc. Urban Plan..

[40] Souza L.C., Giunta M.B. (2011). Urban indices as environmental noise indicators. Comput. Environ. Urban Syst..

[41] Wang B., Kang J. (2011). Effects of urban morphology on the traffic noise distribution through noise mapping: A comparative study between UK and China. Appl. Acoust..

[42] Can A., Leclercq L., Lelong J., Defrance J. (2008). Capturing urban traffic noise dynamics through relevant descriptors. Appl. Acoust..

[43] Wu Y., Kang J., Zheng W. (2018). Acoustic environment research of railway station in China. Energy Procedia.

[44] Pojani D., Stead D. (2015). Transit-Oriented Design in the Netherlands. J. Plan. Educ. Res..

[45] Pojani D., Stead D. (2018). Chapter Four: Past, Present and Future of Transit-Oriented Development in Three European Capital City-Regions. Adv. Transp. Policy Plan..

[46] Kong W., Pojani D. (2017). Transit-Oriented street design in Beijing. J. Urban Des..

[47] Lu Y., Gou Z., Xiao Y., Sarkar C., Zacharias J. (2018). Do Transit-Oriented Developments (TODs) and Established Urban Neighborhoods Have Similar Walking Levels in Hong Kong?. Int. J. Environ. Res. Public Health.

[48] Loo B., Chen C., Chan E. (2010). Rail-Based Transit-Oriented Development: Lessons from New York City and Hong Kong. Landsc. Urban Plan..

[49] Belzer D., Autler G. (2002). Transit Oriented Development: Moving from Rhetoric to Reality.

[50] Cervero R., Ferrell C., Murphy S. (2002). Transit-oriented Development and Joint Development in the United States: a Literature Review.

[51] Kam W., Kalam C., Nancy D., Constantinos S. (2011). Particulate Matter (PM) Concentrations in Underground and Ground-Level Rail Systems in the Los Angeles Metro. Atmos. Environ..

[52] Kang J., Schulte-Fortkamp B. (2017). Soundscape and the Built Environment.

[53] Han M.H., Joo M.K., Oh Y.K. (2010). Residential and Acoustic Environments Perceived by Residents of Regional Cities in Korea: A Case Study of Mokpo City. Indoor Built Environ..

[54] Kim M.J., Braatz R.D., Kim J.T., Yoo C.K. (2015). Economical control of indoor air quality in underground metro station using an iterative dynamic programming-based ventilation system. Indoor Built Environ..

[55] Renne J. (2005). Transit-Oriented Development in Western Australia: Attitudes, Obstacles and Opportunities.

[56] Renne J. (2009). Evaluating Transit-Oriented Development Using a Sustainability Framework: Lessons from Perth’s Network City. Planning Sustainable Communities: Diversity of Approaches and Implementation Challenges.

[57] Noland R., Ozbay K., DiPetrillo S., Iyer S. (2014). Measuring Benefits of Transit Oriented Development.

[58] Ouis D. (2001). Annoyance from Road Traffic Noise: A Review. J. Environ. Psychol..

[59] Seto E.Y.W., Holt A., Rivard T., Bhatia R. (2007). Spatial Distribution of Traffic Induced Noise Exposures in a US City: An Analytic Tool for Assessing the Health Impacts of Urban Planning Decisions. Int. J. Health Geogr..

[60] Xie P., Peng Y., Wang T., Zhang H. (2019). Risks of Ear Complaints of Passengers and Drivers While Trains Are Passing Through Tunnels at High Speed: A Numerical Simulation and Experimental Study. Int. J. Environ. Res. Public Health.

[61] Maclachlan L., Ögren M., van Kempen E., Hussain-Alkhateeb L., Persson Waye K. (2018). Annoyance in Response to Vibrations from Railways. Int. J. Environ. Res. Public Health.

[62] Mao P., Li J., Xiong L., Wang R., Tan Y., Li H. (2019). Characterization of Urban Subway Microenvironment Exposure—A Case of Nanjing in China. Int. J. Environ. Res. Public Health.

[63] Scheer B., Ewing R., Park K., Khan S. (2017). How Does Transportation Affordability Vary Among TODs, TADs, and Other Areas? TREC Final Reports, NITC-RR-859.

[64] Renne J.L., Ewing R. (2013). Transit-Oriented Development: An Examination of America’s Transit Precincts in 2000 & 2010.

[65] Dallas Area Rapid Transit (DART) (2008). Transit-Oriented Development (TOD) Guidelines.

[66] DART (2019). Fact Sheets. https://dart.org/factsheet/default.asp.

[67] Trinity Railway Express (TRE) About Trinity Railway Express 2019. https://trinityrailwayexpress.org/about/.

[68] Center for Hearing and Communication (CHC) (2018). Common Environmental Noise Levels.

[69] Gavin Howard K., Roland-Mieszkowski M., Jason T., Rainham D.G.C. (2012). Noise Levels Associated with Urban Land Use. J. Urban Health.

[70] Piccolo A., Plutino D., Cannistraro G. (2005). Evaluation and analysis of the environmental noise of Messina, Italy. Appl. Acoust..

[71] Gaja E., Gimenez A., Sancho S., Reig A. (2003). Sampling techniques for the estimation of the annual equivalent noise level under urban traffic conditions. Appl. Acoust..

[72] Yao C.M.K.L., Ma A.K., Cushing S.L., Lin V.Y. (2017). Noise exposure while commuting in Toronto—A study of personal and public transportation in Toronto. J. Otolaryngol.-Head Neck Surg..

[73] Loukaitou-Sideris A., Schaffer A. (2014). Too loud to hear the train! Noise assessment, implications, and mitigation strategies on light rail platforms. J. Plan. Educ. Res..

[74] Houston D., Dang A., Wu J., Chowdhury Z., Edwards R. (2016). The cost of convenience; air pollution and noise on freeway and arterial light rail station platforms in Los Angeles. Transp. Res. Part D.

[75] Shimokura R., Soeta Y. (2011). Characteristics of train noise in above-ground and underground stations with side and island platforms. J. Sound Vib..

[76] DART (2016). InMotion—The Official Newsletter of Dallas Area Rapid Transit.

[77] Daly-Standlee Associates (DSA) (2003). Investigation of Dumpster Noise Controls.

[78] Sü Z., Çalıskan M. (2007). Acoustical design and noise control in metro stations: Case studies of the Ankara metro system. Build. Acoust..

[79] California High-Speed Train Project (HST) Eir/Eis (2018). Aesthetics and Visual Resources Merced to Fresno Section.

[80] U.S. DOT Federal Railroad Administration (FRA) (2014). Monitoring Procedure 32C—Project Scope Review.

[81] Dinno A., Powell C., King M.M. (2011). A study of riders noise exposure on bay area rapid transit trains. J. Urban Health.

[82] Gershon R.R.M., Neitzel R., Barrera M.A., Akram M. (2006). Pilot survey of subway and bus stop noise levels. J. Urban Health.

[83] Jacobs F. (2018). Street Grids Matter More to your Commute Than You Might Think.

[84] Rey Gozalo G.B.M.J. (2016). Analysis of sampling methodologies for noise pollution assessment and the impact on the population. Int. J. Environ. Res. Public Health.

[85] Wu H., Kang J., Jin H. (2017). Effects of urban street spatial parameters on sound propagation. Environ. Plan. B Urban Anal. City Sci..

[86] Han X., Huang X., Liang H., Ma S., Gong J. (2018). Analysis of the relationships between environmental noise and urban morphology. Environ. Pollut..

[87] Raudenbush S.W., Bryk A.S. (2002). Hierarchical Linear Models: Applications and Data Analysis Methods.

[88] Field A.P. (2005). Discovering Statistics Using SPSS.

[89] van Kempen E., Devilee J., Swart W., van Kamp I. (2014). Characterizing urban areas with good sound quality: Development of a research protocol. Noise Health.

[90] Korpela K., Ylen M., Tyrvainen L., Silvennoinen H. (2009). Stability of self-reported favourite places and place attachment over a 10-month period. J. Environ. Psychol..

[91] Evans G.W. (2003). The built environment and mental health. J. Urban Health.

[92] Booi H., van den Berg F. (2012). Quiet areas and the need for quietness in Amsterdam. Int. J. Environ. Res. Public Health.

[93] Yu W.L., Kang J. (2017). Relationship between traffic noise resistance and village form in China. Landsc. Urban Plan..

[94] Caltrans (2011). Traffic Noise Analysis Protocol for New Highway Construction, Reconstruction and Retrofit Barrier Projects.

[95] Seidman M.D., Standring R.T. (2010). Noise and quality of life. Int. J. Environ. Res. Public Health.

[96] Zambon G., Benocci R., Bisceglie A., Roman H.E., Smiraglia M. DYNAMAP project: Procedure for noise mapping updating in urban area. Proceedings of the INTER-NOISE and NOISE-CON Congress and Conference.

[97] Cervero R., Murphy S., Ferrell C., Goguts N., Tsai Y.-H., Arrington G.B., Boroski J., Smith-Heimer J., Golem R., Peninger P. (2004). Transit-Oriented Development in the United States: Experiences, Challenges, and Prospects.

